# Robust detection for selective harvesting of field flat jujube: overcoming occlusion and small-target challenges in unstructured environments

**DOI:** 10.3389/fpls.2026.1795650

**Published:** 2026-05-11

**Authors:** Shilin Li, Shangjian Guo, Sheng Gao, Lili Sun, Fuzhong Li, Shujuan Zhang

**Affiliations:** 1 College of Software, Shanxi Agricultural University, Jinzhong, China; 2 College of Agricultural Engineering, Shanxi Agricultural University, Jinzhong, China

**Keywords:** agricultural engineering, automated picking, computer vision, contextual information, selective picking, separated and enhancement attention, small target detection

## Abstract

The detection of field flat jujube is constrained by their characteristics and the complex agricultural environment, presenting challenges such as small target size and dense occlusion, which can easily lead to insufficient information in occluded areas and the loss of features in small targets. This paper builds upon the latest YOLOv12 (You Only Look Once) network, focusing on compensating for information loss in occluded regions and improving detection accuracy for small objects. Firstly, the Separated and Enhancement Attention Module (SEAM) was incorporated into the neck network to enhance feature representation in occluded regions. Secondly, to enrich contextual semantic information in dense detection tasks, we replaced the up-sampling operator with the Content-Aware Reassembly of Features (CARAFE) operator. Finally, the Parallelized Patch-aware Attention (PPA) module was integrated into the detection head to design a small-target-specific detection head with a built-in attention mechanism, through which the interactive fusion of global and local feature representations was realized. Experimental results demonstrated that the proposed YOLOv12-SCP network achieved a mean average precision (mAP@0.5) of 96.8% and an F1-score of 0.91, surpassing the original YOLOv12n model by 1.2% and 1.0%, respectively. Meanwhile, the mAP@0.5:0.95 increased by 2.7 percentage points, with the average precision for ripe and unripe fruits reaching 97.6% and 96%, respectively. Through extensive ablation experiments and comparisons with current mainstream object detection methods, it is demonstrated that this method exhibits superior performance in detecting small object occlusions in complex environments.

## 1 Introduction

Robust detection of flat jujube in natural orchard environments remains challenging due to complex backgrounds, dense occlusion, and small target scales substantially degrade the performance of existing object detection methods. The flat jujube, a representative flat fruit, exemplifies this dual challenge: its small size relative to harvesting scenarios involving 2–4 m tall, round-canopied trees renders it particularly susceptible to simultaneous small target detection and occlusion overlap. Small objects suffer from the issue of scarce features or even being overwhelmed by noise due to their limited pixel count, while general-purpose object detection models are often optimized for medium or large objects, resulting in weaker perception capabilities for small targets. Simultaneously, occlusion compromises the structural integrity of objects, resulting in the loss of key features and even leading to semantic confusion between the target and background. When small-scale objects and partial occlusion co-occur, model performance typically degrades significantly. This limitation forms a key bottleneck that constrains the accuracy of existing detection algorithms, severely impairing the practical applicability and operational precision of harvesting robots in complex field environments. Robust detection of small and occluded objects in the field is both a fundamental computer vision challenge and a practical requirement for agricultural automation.

Notably, most current research on fruit detection relies on publicly available datasets or images collected in controlled greenhouse environments. For flat jujube detection in the field, however, no publicly accessible dataset exists that captures the genuine complexity of real-world agricultural scenes, where harsh sunlight, dense leaf occlusion, and clusters of small, overlapping fruits are the norm. This lack of a dedicated dataset is in itself a major barrier to practical deployment in this domain. To address this challenge, we first construct a flat jujube detection dataset containing 11240 high-resolution field images. The dataset covers different growth stages and spans the full range of natural lighting conditions throughout the day, providing a realistic and demanding benchmark for algorithm evaluation. Building on this foundation, we then examine how a robust network model can be adapted to the specific characteristics of this dataset.

In complex, unstructured agricultural environments, overlapping occlusion and small target recognition constitute the primary challenges compromising detection accuracy and efficiency. Traditionally, the focus of target detection has been on the features in non-occluded areas rather than features in obstructed areas, particularly those that may only arise under more complex environment (Li et al., 2022). With the increasing adoption of computer vision in smart agriculture, image-based automatic detection of field-grown produce has become an essential technology for crop monitoring, automated harvesting, and post-harvest handling (Ariza-Sentís et al., 2024). YOLO networks have advanced rapidly in the last decade, with research improvements focusing on scenario-specific enhancements to detection accuracy and efficiency (Li et al., 2025). Recent years have seen considerable progress in lightweight object detection tailored for resource-constrained or domain-specific scenarios. For instance, YOLOv5-based detectors have been effectively deployed for identifying insect pests in agricultural fields (Ahmad et al., 2022), while more recent architectures employ dynamic feature fusion (Ahmad et al., 2025) or edge-aware representations (Lu et al., 2025) to handle small targets and degraded image quality. These developments highlight a clear trend toward lightweight, task-specific model engineering. Our work follows this direction, focusing specifically on the unique visual challenges of flat jujube detection in field environments.

Occlusion problem. In agricultural settings, the crisscrossing distribution of branches and the disorderly growth of leaves are inevitable. Additionally, the presence of other objects such as shed poles and hanging branch ropes can cause interference, which is highly likely to lead to missed detection and false detection. In order to address the loss of features in occluded areas, Hu et al. (2025) integrated Mamba and DySample structures into the neck network, enhancing multi-scale feature extraction and fusion capabilities. An alternative approach focuses on enhancing feature extraction capabilities through architectural improvements. Xiao et al. (2025) employed a dual-backbone structure to capture the features of occluded fruits instances, achieving an accuracy of 88.1% under severe occlusion conditions. Also based on dual-backbone of Convolutional Neural Network (CNN), Xiong et al. (2025) employed the dual attention mechanism and dynamic convolution to supplement the missing features in occluded regions. Additionally, some researchers integrate the prediction module with a tailored loss function, designed to suppress feature degradation caused by boundary discontinuity. Wu et al. (2025) integrated an angle prediction module with circular smooth labels and a minimum point distance IoU loss and achieved 81.1% accuracy via multistage transfer learning. Research indicates that contextual information enhances scene comprehension when isolated object recognition is challenging (Jamali et al., 2025). To enhance contextual information extraction, Chen J. et al. (2024) integrated the ConvNeXt V2 backbone with the Multi-Scale Dilated Attention (MSDA) module, thereby improving the network’s capability for recognizing multi-scale and arbitrarily-shaped targets. Wang B. et al. (2025) developed a Mixed-Scale Network (MSNet) and implemented a scaling strategy to leverage mixed-scale contextual information, enhancing neural network representational capacity. The aforementioned approaches collectively seek to mitigate issues such as feature loss and degradation caused by occlusion from multiple perspectives.

Small object detection problem. The detection of small target fruits often faces challenges due to insufficient feature information, making them difficult to detect. Therefore, to mitigate limited semantic information in small targets, Kiobya et al. (2025) introduced a multi-scale semantic information enhancement module to infuse fine-grained details from higher prediction layers into the dedicated lowest layer for small target detection. However, this method exhibits insufficient sensitivity to small object features, Liu et al. (2025) added a surface feature output layer to mitigate information loss during successive convolutional operations. In addition, some scholars have conducted research specifically on attention mechanisms for small target detection (Ding et al., 2025; Pu et al., 2025). For instance, Wang P. et al. (2025) integrated the LCE module and the CAE module to achieve feature enhancement in potential target regions, thereby guiding the network towards enhanced focus on small target features. Another direction for improvement focuses on network architecture. For instance, Liu et al. (2025) added a surface feature output layer to prevent the loss of small object features during successive convolutions, which yielded an improvement of 3.7% in mAP@0.5. Similarly, Wang H. et al. (2025) proposed a lightweight cross-layer output reconstruction module, which enhances the integration of shallow and deep feature information via cross-layer connections, achieving a 3.2% increase in accuracy. Moreover, contextual information proves similarly instrumental for small object detection. Yang et al. (2025) developed a dynamic context-aware aggregation strategy designed to prune redundant semantic features, enabling the effective capture of contextual and structural cues for small targets and boosting the detection rate by as much as 7.2 percentage points.

This work presents a multi-module, coordinated solution designed specifically for the task of flat jujube detection in field environments. The approach integrates established components (including SEAM, CARAFE, and PPA) into the YOLOv12n framework to directly address the challenges posed by occlusion and small object detection in real agricultural settings, in which the highlighted contributions are as follows

1. We construct a flat jujube detection dataset designed for real field conditions. Capturing the challenges of variable outdoor lighting, heavy occlusion, and small fruit targets, this large-scale, carefully annotated dataset offers a scarce yet essential benchmark for advancing computer vision research in agricultural field settings.
2. Given the frequent and heavy occlusion typical of flat jujube scenes, the SEAM structure was introduced in the neck network to enhance the feature response in non-occluded regions while compensating for information loss in occluded areas, thereby enhancing the detection effectiveness of occluded targets; To address the feature information loss caused by the dense cluster growth of fruits, the CARAFE operator was used for feature up-sampling to further enrich the contextual feature information;
3. Based on the principles of multi-branch feature extraction and self-attentive feature fusion, we integrated the PPA module into the detection head, enhancing the recognition capability for small targets.

The work’s technical contribution lies in demonstrating how this particular combination of modules effectively addresses the unique, compounded visual challenges (high occlusion, small scale) presented by our newly constructed dataset. Experimental results show that this task-specific integration yields improved detection accuracy and greater robustness when evaluated on the field dataset constructed in this study.

## 2 Materials and methods

### 2.1 Construction of data set

**Figure 1:**
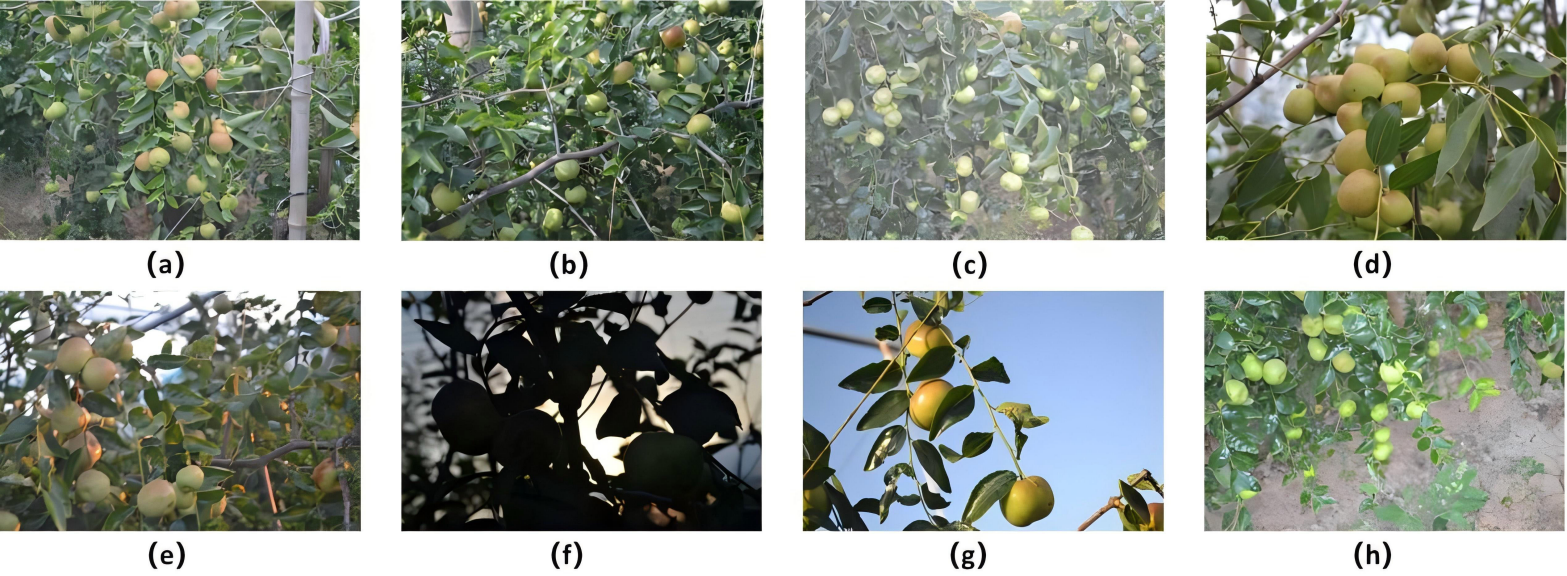
The data set of field flat jujube. **(a)** occlusion - columns **(b)** occlusion - branches **(c)** occlusion - leaves **(d)** occlusion - overlap **(e)** front lighting **(f)** backlight **(g)** background - sky **(h)** background - land.

This study focused on the jujube cultivar ‘Youpan’, a flat-fruited variety. Fruit samples were collected during the expansion and ripening stages for subsequent analysis. RGB images of field flat jujube were collected in Shangguo Township, Yanhu District, Yuncheng City, Shanxi Province (110.65°E, 35.05°N) between August 28 and September 15, 2023. The image acquisition employed a Nikon D3100 camera and an Honor mobile phone, with a focal length of 22 mm and a shooting distance ranging from 40 cm to 80 cm. Images were captured between 08:00 and 19:00, yielding a total of 11,240 digital RGB images at a resolution of 3456 × 2304 pixels. The data collection process included not only common obstructions such as leaves, branches, and fruits, along with minor obstructions (e.g., shed poles and branch-tying ropes). Photographs were captured under both backlight and front-lighting conditions across diverse weather scenarios. Partial data is presented in **Figure 1**.

In this experiment, the LabelImg 1.8.6 (Tzutalin, Canada) tool was used to manually annotate the data set (11,240 images, 67,846 bounding boxes) with categories mature jujube (48,321) and immature jujube (19,525). The data set was randomly partitioned in an 8:1:1 ratio with no overlap, comprising a training set (8,992 images; 57,072 bounding boxes), a validation set (1,124 images; 5,605 bounding boxes), and a test set (1,124 images; 5,169 bounding boxes).

### 2.2 Model infrastructure

Driven by advancements in computing and mobile hardware, and considering the requirements for algorithm application and deployment, this research focuses on compact models, specifically the n and tiny sizes. After reviewing the GitHub (official code hosting platform) and related literature, partial data of various benchmark networks on the COCO official data set (NVIDIA V100 GPU) are presented in **Table 1**.

**Table 1:** Some data of each basic network on the COCO official data set.

Model	mAP@0.5(%)	FPS(ms)	Params(M)	Size(MB)	FLOPs(G)
YOLOv3-tiny	18.9	200+	8.7	17.6	5.6
YOLOv4-tiny	21.7	180+	6.0	12.1	6.8
YOLOv5n	31.4	280+	1.9	3.8	4.5
YOLOv6n	35.1	120+	4.7	9.1	11.1
YOLOv7-tiny	38.7	95+	6.0	12.1	13.2
YOLOv8n	37.3	130+	3.2	6.2	8.7
YOLOv9-tiny	38.3	——	2.0	4.4	7.7
YOLOv10n	38.5	Latency=1.84ms	2.3	10.9	6.7
YOLOv11n	39.5	T4 TensorRT10 = 1.5ms	2.6	5.35	6.5
YOLOv12n	40.6	T4 TensorRT10 = 1.6ms	2.5	5.33	6.0

YOLOv12 (Tian et al., 2025), the latest advancement in detection frameworks, uniquely centers on attention mechanisms to harness the transformer’s advantages for enhanced real-time computational efficiency. Based on the content of the table above, and taking into account the complexity of agricultural environments and the characteristics of flat jujube, which are highly susceptible to small object detection and overlapping occlusion issues, we have selected YOLOv12n as the baseline model due to its highest accuracy and relatively superior model size advantage. It retains a conventional architecture comprising an input module, backbone network, neck network, and detection heads. The input end is responsible for receiving training data and performing data augmentation. The backbone network, primarily composed of conv, A2C2f, and C3k2 modules, is tasked with feature extraction from images. Multiple convolutional modules perform essential feature extraction through convolution, batch normalization, and activation functions. The C3k2 module is primarily used to assist in feature extraction, reducing redundant computations through parallel convolutional branches, thereby enhancing the efficiency of feature extraction. Integrating the flash attention mechanism from the Area Attention (A2) module and Residual Efficient Layer Aggregation Network (R-ELAN) concepts, the A2C2f module minimizes parameters and computational load, thus improving model efficiency. The neck network fully leverages up-sampling and concatenation operations to fuse backbone-extracted features by integrating multi-level information for enhanced feature representation. Its overall structure is primarily based on FPN+PAN, including concatenation layers, up-sampling layers, and the A2C2f module. The head component at the output end is responsible for classifying and detecting the processed feature maps.

### 2.3 Improvement of the YOLOv12

#### 2.3.1 Separated and enhancement attention module

**Figure 2:**
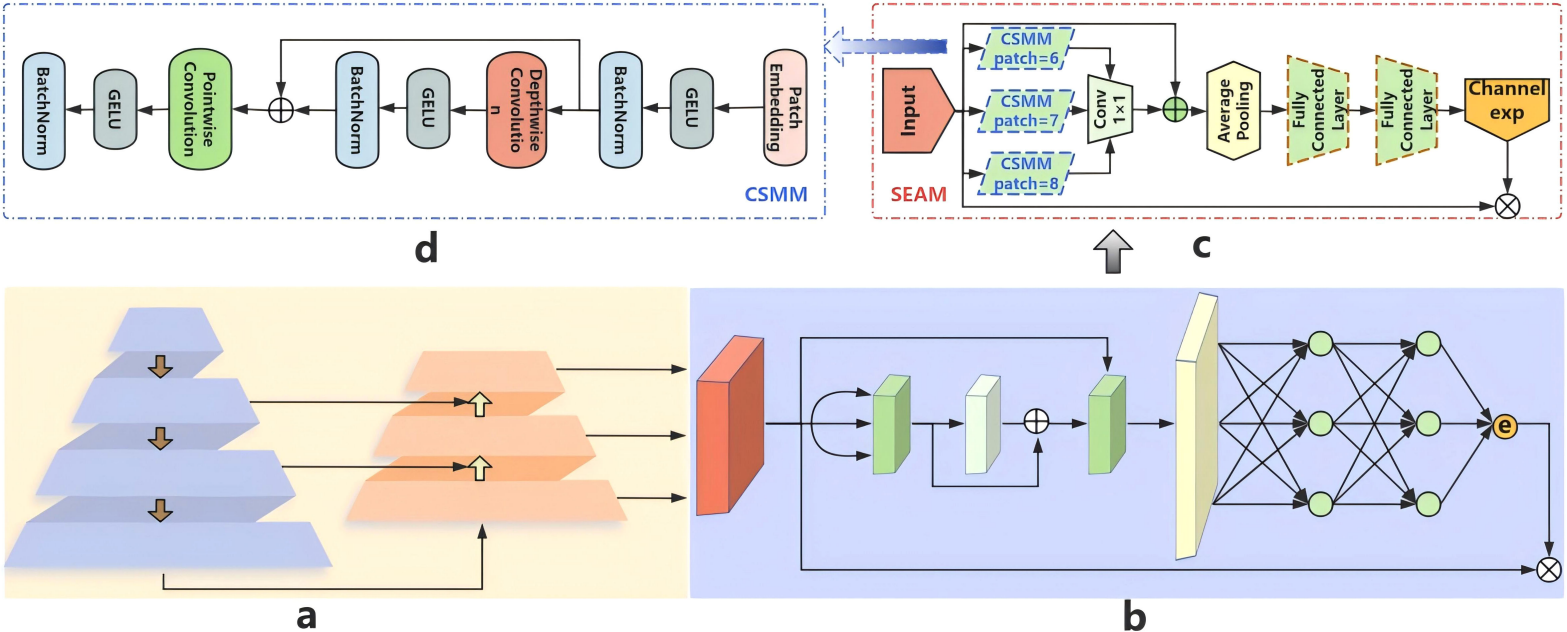
The structure diagram of SEAM. **(a)** neck: FPN+PAN **(b)** the process. Of the SEAM Module **(c)** the architecture of the SEAM. **(d)** the structure of CSMM.

In complex agricultural environments, flat jujube are prone to occlusion and overlapping, which can lead to alignment errors, local aliasing, and feature loss issues. Traditional methods may result in decreased detection accuracy due to feature loss. To address this issue, we introduce the SEAM (Yu et al., 2024) module to improve the model’s capability to detect occluded targets. The SEAM module used in this study is specifically modified for the field flat jujube detection scenario in structural simplification. The design of this module primarily aims to achieve three objectives: enabling multi-scale object detection, enhancing the features of fruit regions in images, and relatively weakening the background regions, with the specific structure illustrated in **Figure 2**.

**Figure 2a** depicts the neck network integrating Feature Pyramid Network (FPN) and Path Aggregation Network (PAN) architectures. **Figure 2b** illustrates the SEAM module positioned after the neck’s output layer, which leverages inter-feature relationships to recover occluded feature information. **Figure 2c** shows the complete architecture of the SEAM module, with its most crucial part being the Channel and Spatial Mixing Module (CSMM) in **Figure 2d** (denoted by the blue dashed box in **Figure 2c**). The patch settings for the 3 parallel branches in the CSMM of this paper is adjusted from 3, 5, 7 to 6, 7, 8. This modification is made for two reasons: first, to expand the receptive field and enhance the reasoning capability for contextual information, thereby compensating for features lost in occluded areas; second, to maintain scale continuity and enable denser scale sampling. It processes multi-scale features using patches of different sizes and employs depth-wise separable convolutions with residual connections (the red part in **Figure 2d**) to learn spatial-channel correlations.

While effectively capturing channel importance and reducing parameters, this architecture neglects cross-channel correlations. We remedy this by integrating multi-depth convolution outputs through 1×1 point-wise convolutions (the green part in **Figure 2d**). A two-layer FC network (the brown dashed box in **Figure 2c**) is then employed to integrate information across channels, thereby enhancing the correlations among all channels. The SEAM model aims to compensate for the aforementioned information loss in occluded scenarios by leveraging the relationships between occluded and non-occluded fruits learned in the previous stage. Exponential transformation of FC layer outputs converts [0,1] activations to [1,e], establishing monotonic normalization that mitigates localization error sensitivity. Finally, the SEAM module’s output weights the original features as attention weights, enabling more effective handling of overlapping targets. This mechanism enhances feature response in non-occluded regions while recovering information in occluded areas, thereby improving occluded object detection. Consequently, we integrate SEAM into the neck network to boost occlusion detection capability for flat jujube.

#### 2.3.2 Content-aware reassembly of features

**Figure 3:**
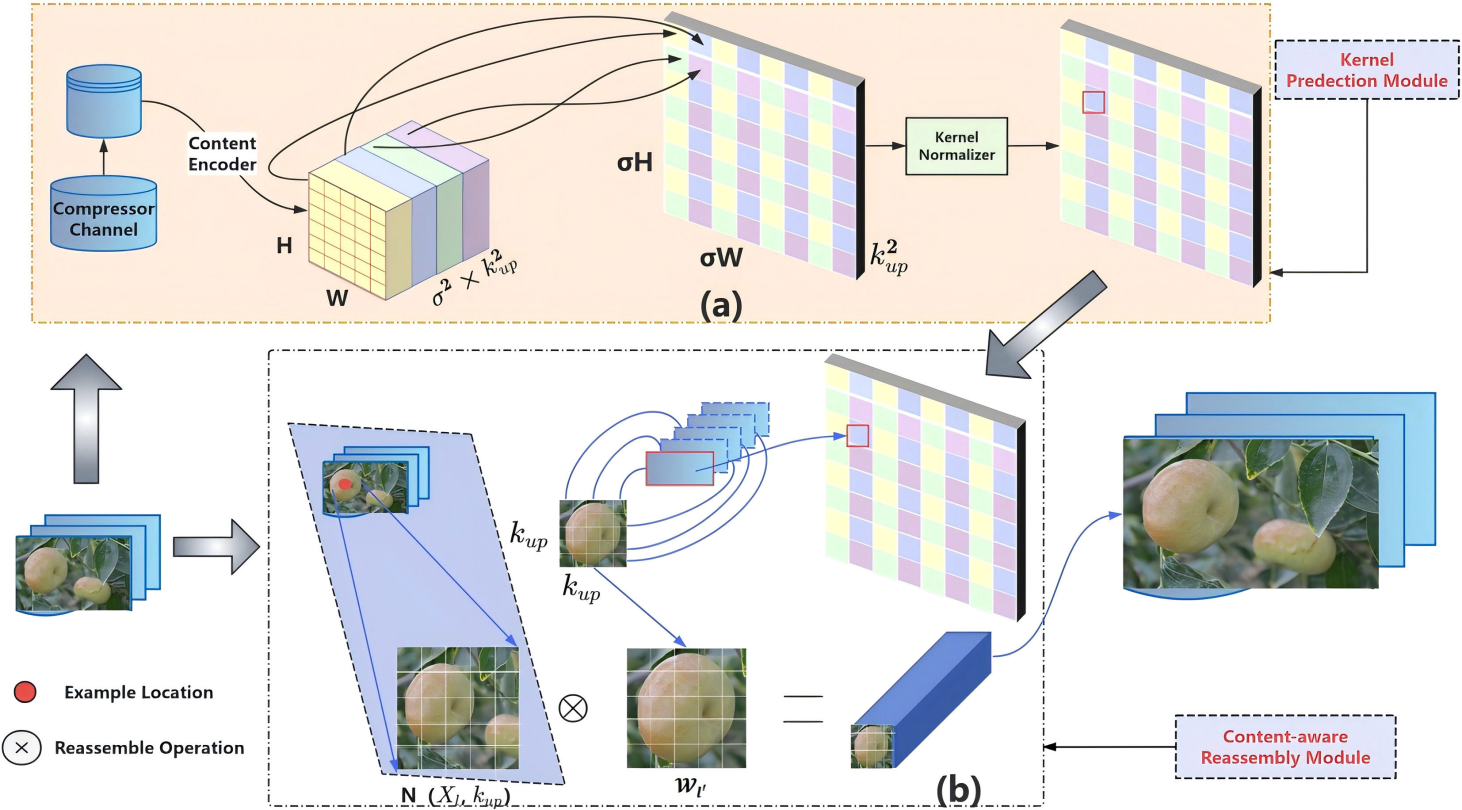
The overall framework of CARAFE. CARAFE is composed of two key components: **(a)** kernel prediction module **(b)** content-aware reassembly module. A feature map with size C × H × W is up-sampled by a factor of (σ= 2) in this figure.

Feature up-sampling in the YOLO algorithm is typically located in the neck network, serving a critical function in the FPN section by primarily facilitating the fusion of low-resolution and high-resolution feature maps. Traditional up-sampling methods have two main drawbacks: (1) They can only utilize contextual information within a small neighborhood, e.g., bilinear interpolation and nearest neighbor interpolation. (2) They incur high computational costs for calculating adaptive interpolation, e.g., deconvolution. To this end, we adopt the CARAFE up-sampling operator (Wang et al., 2019), which aggregates contextual information within large receptive fields, dynamically adapts to specific content, and ensures computational efficiency. The details of its structure are presented in **Figure 3**.

The kernel prediction module (**Figure 3a**) integrates three sequential sub-modules: (1) a channel compressor reducing input feature map dimensions; (2) a content encoder processing compressed features to generate reassembly kernels; (3) a kernel normalizer applying softmax operations per kernel. **Figure 3** illustrates the working principle of the CARAFE operator: following up-sampling, the feature map more accurately represents object shapes (purple parallelogram in **Figure 3b**), consequently improving model predictions. CARAFE reorganizes features within a predefined region centered at each position via weighted aggregation, where weights are adaptively generated by a lightweight fully convolutional module with softmax activation (green module in **Figure 3a**) in a content-aware manner. The content-aware reorganization module (**Figure 3b**) will reorganize the features within local regions using the function ϕ. By assigning higher weights to relevant feature points within local regions, the reorganized features exhibit enhanced representational strength compared to the original features.

The process comprises two sequential stages:

1. Content-aware kernel prediction: For each target location l’, the kernel prediction module ψ generates a location-wise reassembly kernel W_v_ based on the neighborhood of X_l_, as shown in Equation 1.

(1) $${\text{W}}_{\text{v}}=\text{ψ}(\text{N}({\text{X}}_{\text{l}},{\text{k}}_{\text{encoder}}))$$

2. Feature reassembly: The content-aware module ϕ reconstructs features by aggregating the neighborhood of X_l_ using the predicted kernels W_v_, as shown in Equation 2.

(2) Xl''=ϕ(N(Xl,kup),Wv)

Given an input feature map X of size C × H × W and an integer upsampling ratio σ, CARAFE generates an output feature map X’ of size C × σH × σW. For each target location l’= (i’, j’) of the output X’, there exists a corresponding source location l = (i, j) in X. The k×k neighborhood centered at l is denoted as N (X_l_, k).

**Figure 4:**
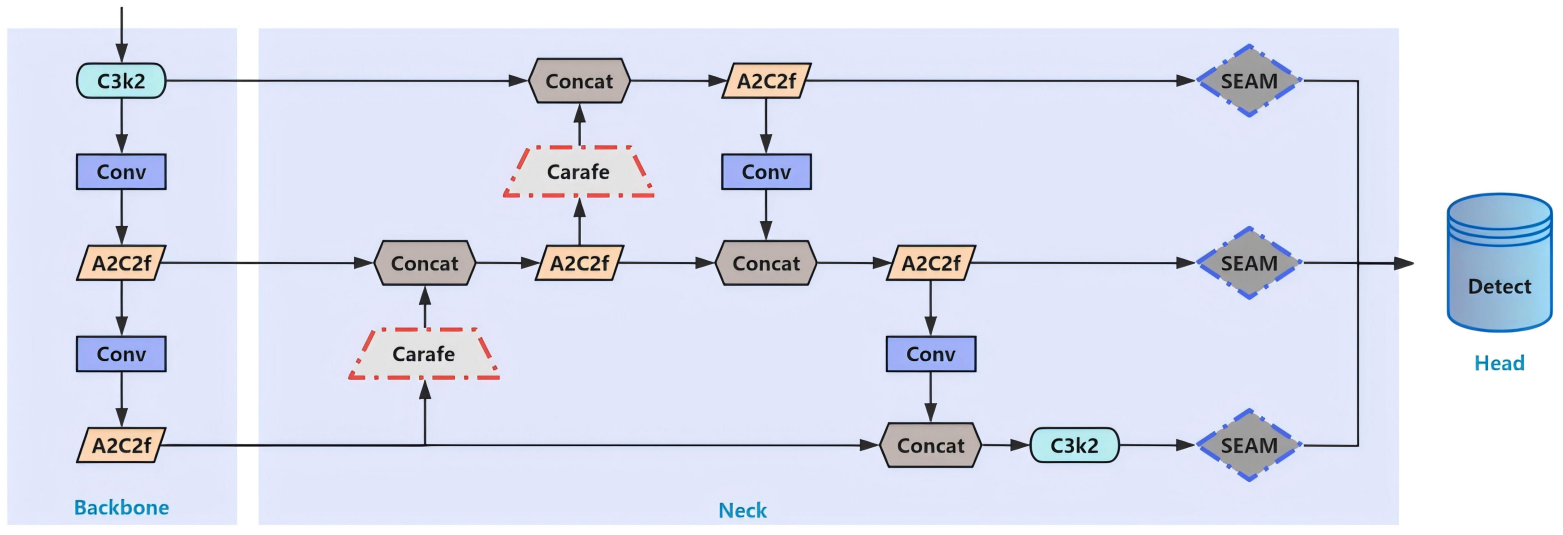
Improvement locations for each module. CARAFE: red dashed line section. SEAM: blue dashed line section.

The specific improvement locations of each module are shown in **Figure 4**.

#### 2.3.3 Parallelized patch-aware attention module

**Figure 5:**
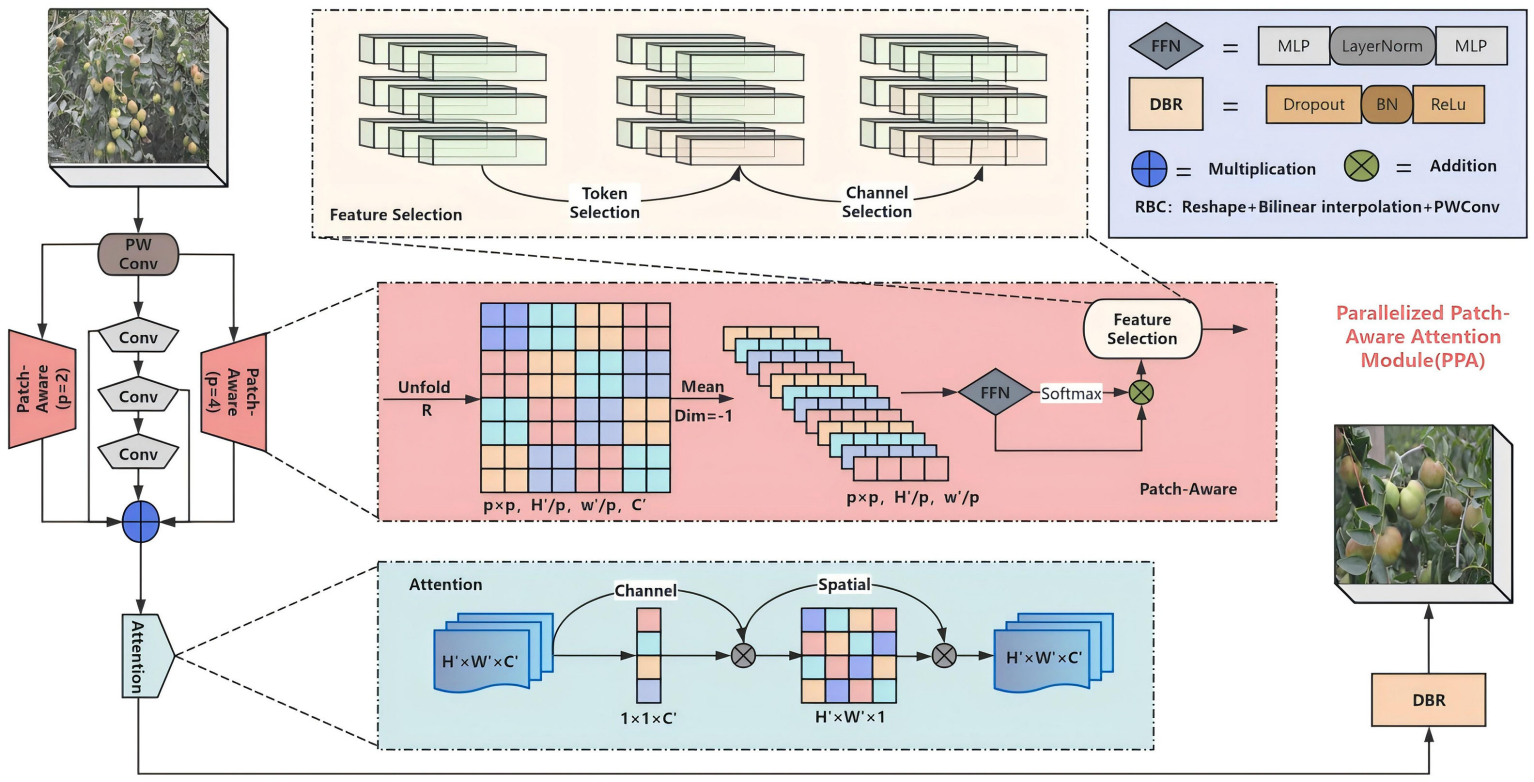
Detailed structure of the PPA module. Components: multi-branch fusion and attention mechanism. Among them, the multi-branch fusion part includes patch-aware and concatenated convolution. The ‘p’ parameter in patch-aware is set to 2 and 4, corresponding to the local branch and the global branch, respectively.

YOLOv12, being the only YOLO network with a self-attention mechanism, inherently captures key information of small objects more accurately. To further augment small object feature extraction capacity, we incorporate the attention-based PPA module (Xu et al., 2024) into the detection head. The details of structure are presented in **Figure 5**.

As illustrated in **Figure 5**, PPA utilizes a parallel multi-branch architecture where each branch extracts hierarchical features at distinct scales. This strategy enhances differentiation between small targets and complex backgrounds through multi-scale feature representation learning. This multi-branch strategy includes three parallel branches: a local convolution branch (the red trapezoid on the left, p=2), a global convolution branch (the red trapezoid on the left, p=4), and a sequence convolution branch (the gray pentagon on the left).

The processing pipeline operates as follows: given a feature tensor (F∈H’, W’, C), three convolutional branches (local, global, and serial) process the tensor. Firstly, point-wise convolution (PW Conv) transforms F into (F’∈H’, W’, C’). Then, three branches are used to compute F’_local, F’_global, and F’_conv respectively. Finally, through a series of operations, the features are selected and their weights are adjusted. The results from the three branches are then summed to obtain the fused features, which are passed into the attention mechanism to achieve adaptive feature enhancement. The attention module combines an efficient channel attention mechanism with a spatial attention component. Features first pass sequentially through the 1D channel attention and 2D spatial attention mechanisms. Subsequent processing involves activation functions, batch normalization, and dropout operations to generate the final output.

The advantages of multi-branch feature extraction: this strategy can capture multi-scale features of objects, thereby improving the accuracy of small target detection. Simultaneously, different branches can focus on information at multiple scales and levels, reducing the feature loss caused by repeated down-sampling at a single scale. The advantages of feature fusion and attention mechanisms: the attention mechanism adaptively augments feature representational capacity, thereby accentuating critical small-target information. The synergistic integration of channel and spatial attention mechanisms discriminatively enhances small-target features, thereby augmenting the network’s representational capacity for small objects.

### 2.4 Experimental environment and parameter setting

Experiments were conducted on a Windows 10 platform with the following hardware configuration: AMD R7-5800H CPU (8 cores/16 threads, 4.4GHz max frequency), 16GB RAM, 3TB SSD, and NVIDIA GeForce RTX 3070 GPU (8GB VRAM). The software environment utilized Python 3.9.1, PyTorch 1.11.0, CUDA 11.3, and cuDNN 8.1.1, with development in PyCharm 2019. All models were trained on identical datasets, with hyperparameter configurations specified in **Table 2**.

**Table 2:** Training hyperparameter configurations.

Hyperparameter	Value	Hyperparameter	Value
Epochs	100	Optimizer	SGD
Batch Size	32	Amp	True
Image Size	640×640	Lr	0.01
Workers	4	Momentum	0.937

### 2.5 The indicators of evaluation

The evaluation metrics adopted in this study were primarily divided into two major aspects: accuracy and complexity assessment. Model accuracy metrics: Precision (P), Recall (R), mean Average Precision (mAP), and F1 score. Among these, P was used to evaluate the precision of the predictive model, while R was used to measure the model’s recall. The comprehensive indicator of precision and recall was represented by the mAP, with specific calculation formulas shown in Equation 3 and Equation 4:

(3) $$\text{P}=\frac{\text{TP}}{\text{TP}+\text{FP}}\times 100\%,\text{R}=\frac{\text{TP}}{\text{TP}+\text{FN}}\times 100\%,\text{F}1=\frac{2\times \text{P}\times \text{R}}{\text{P}+\text{R}}$$

(4) $$\text{AP}=\int_{0}^{1}\text{P}(\text{R})\text{dR},\text{mAP}=\frac{1}{\text{s}}\sum_{\text{i}=1}^{\text{s}}{\text{AP}}_{\text{i}},$$

Where TP denotes true positives (correctly detected fruits), FP signifies false positives (detected but not fruits), and FN represents false negatives (undetected but actually existing fruits), with s indicating the category count.

Model complexity was evaluated using three metrics: Parameters, giga floating-point operations per second (GFLOPs), and model size. The corresponding calculations are shown in Equations 5–7:

(5) $$\left\{ \begin{array}{c} Parameters(Conv)=C_{out}\times (K^{2}\times C_{in}+1)\\ Parameters(FC)=(n_{in}\times n_{out})+n_{out} \end{array}\right.$$

(6) $$\left\{ \begin{array}{c} FLOPs(Conv)=(2\times C_{in}\times K^{2}-1)\times H\times W\times C_{out}\\ FLOPs(Pool)=\frac{1}{S}(H\times W\times C_{out})\\ FLOPs(FC)=2\times B\times C_{in}\times C_{out} \end{array}\right.$$

(7) $$\text{Model Size}=\frac{1}{1024^{2}}(4\times \text{parameters})$$

Where C_in_, K, and C_out_ denote the input channel count, convolution kernel size, and output channel count, respectively. H×W represents the output spatial size; B indicates the batch size; S refers to the convolution stride. n_in_ and n_out_ correspond to the input and output node counts.

## 3 Experimental results and analysis

In this chapter, we conducted comprehensive ablation experiments on the proposed method, including the effectiveness of the attention module, the optimization of the up-sampling operator, and the design of the detection head. Subsequently, we compared the comprehensive performance of the proposed algorithm with other mainstream object detection algorithms. To ensure a fair and controlled comparison, all baseline detectors and the proposed method were trained and evaluated under identical conditions. Specifically, the dataset split, input resolution (640×640), data augmentation pipeline, optimizer (SGD with lr=0.01, momentum=0.937, weight decay=5e-4), and evaluation metrics were held constant across all experiments.

### 3.1 Effects and analysis of different attention modules and structures on feature fusion

We aim to minimize the impact of such irrelevant information and focus more on the target’s data. We selected relatively new methods from the past 3 years to improve the neck network, primarily incorporating the Attention Scale Sequence Fusion (ASF) module, Channel Transposed Attention (CTA) module, Semantics and Detail Infusion (SDI) module, and the SEAM module into the neck network. The SEAM module includes three parallel branch schemes with convolution kernel sizes of (3,5,7), (6,7,8), and (5,7,9) respectively. Additionally, we attempted to replace the neck network structure with the High-level Screening-Feature Pyramid Network (HS-FPN) structure for comparative analysis, and selected several combination schemes that could enhance accuracy, as shown in **Table 3**.

**Table 3:** The influence of different attention modules and structures.

Module	P(%)	R(%)	mAP@0.5(%)	mAP@0.5:0.95(%)	F1 (%)	Parameters	FLOPs(G)	Size (MB)
YOLOv12n	92.6	88.3	95.6	81	90	2520054	6.0	5.4
+ASF	90.5	87.3	95.2	79.5	89	2881857	7.9	6.2
+CTA	89.9	87.6	94.9	78.2	89	3196034	6.9	6.7
+SDI	92.2	88.1	95.8	81.0	90	2618070	7.0	5.7
+SEAM(3,5,7)	89.1	86.2	94.2	77.4	88	2403446	5.6	4.6
+SEAM(6,7,8)	92.5	89.4	96.1	81.8	91	2653494	6.6	5.7
+SEAM(5,7,9)	88.9	89.0	95.3	79.4	89	4183470	7.4	8.7
+HS-FPN	90.9	88.2	95.6	79.5	89	1871030	6.5	4.1
SDI+ASF	92.2	88.1	95.8	81.0	90	3589110	8.3	7.6
SEAM+SDI	91.7	88.8	96	81.2	90	2681590	6.6	5.7

**Figure 6:**
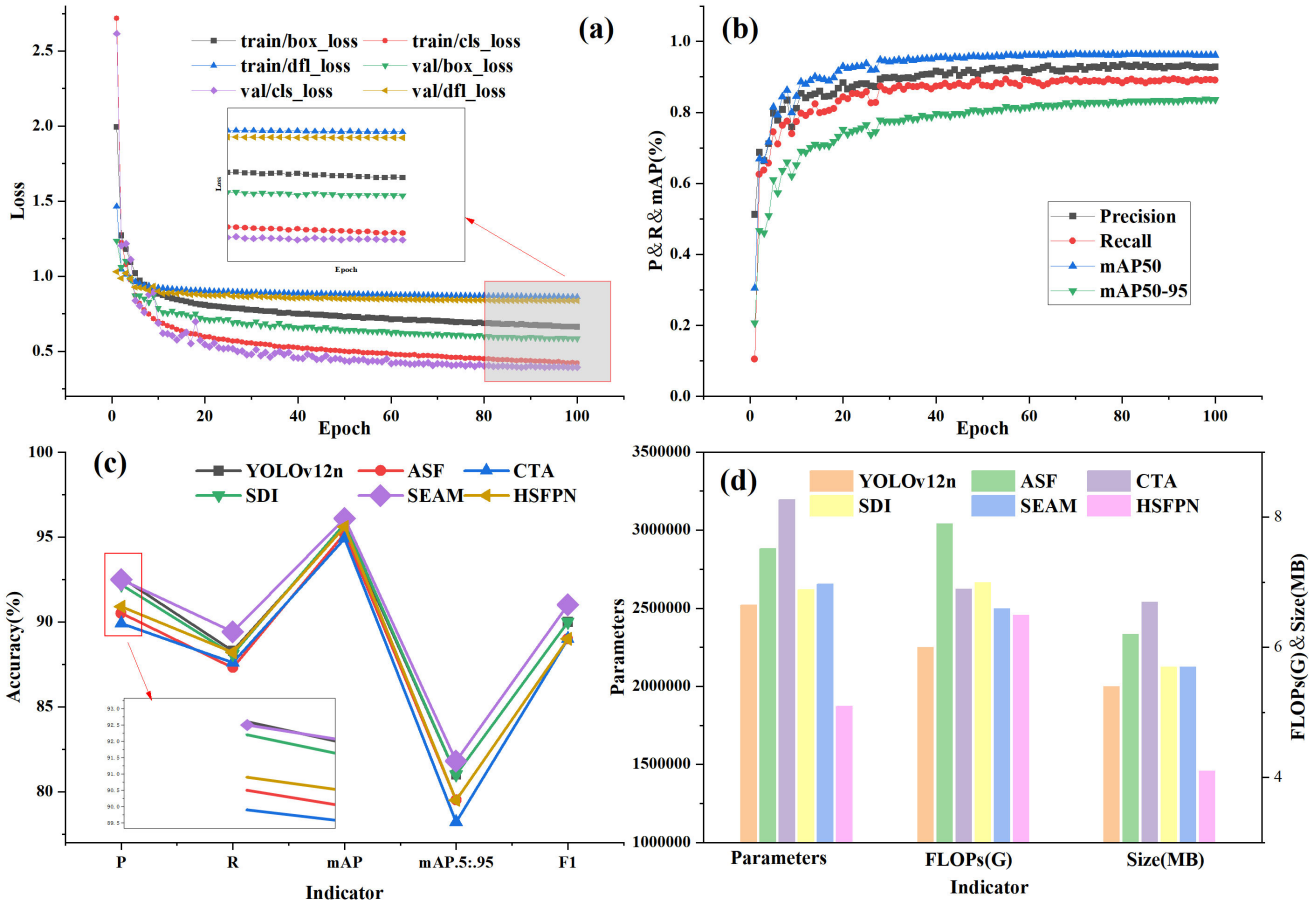
The influence of different attention modules and structures. **(a)** and **(b)** are the loss curve and accuracy curve of the improved network, **(c)** and **(d)** are the comparisons of accuracy and complexity between the five improved modules and the baseline network.

From the data in the table above, it can be observed that although the ASF (Kang et al., 2024) and CTA (Dai et al., 2024) modules excel in fusing multi-scale features, their accuracy in detecting occluded fruits is relatively low. The SDI (Peng et al., 2025) module also serves to suppress background and other interfering factors, highlighting the key features and positional information of the target. This module can also bring improvements in accuracy metrics, but the overall increase is not significant (+0.2%). Meanwhile, compared to SEAM, it has disadvantages in terms of parameter count and detection time. The HS-FPN (Chen Y. et al., 2024) structure enhances the detection of subtle features by employing a multi-scale fusion strategy to address the issue of feature scarcity and reduce the loss of features in occluded regions. It decreased the parameters and model size by 25.75% and 24.07%, respectively. Although it can make the model more lightweight, the precision loss is too significant. Among the three SEAM schemes, the convolution kernel sizes (3×3) and (9×9) are set for minimal and maximal targets respectively, which respectively increase the missed detection rate and computational cost, resulting in fusion conflicts. Most notably, the SEAM (6,7,8) approach achieves peak values of 96.1, 81.8, and 91 for mAP@0.5, mAP@0.5:0.95, and F1, respectively. This performance is attributed to its contextual reasoning capabilities and dense, continuous multi-scale extraction. The specific trends of each data are shown in **Figure 6**.

As can be seen from **Figure 6a, b**, the loss and accuracy curves of the improved network achieve convergence at 50 epochs, with no overall fluctuations. From the blue histogram in **Figure 6d**, it is evident that the three metrics show slight increases (Parameters +5.29%, Model Size +10%, FLOPs +5.56%, with the second smallest increase compared to other methods). This stems from the SEAM module necessitating additional computational layers to perform feature separation, enhancement, and fusion operations, thereby increasing network structural complexity.

But most importantly, the SEAM module achieved mAP@0.5 and mAP@0.5:0.95 of 96.1% and 82.4% respectively, showing an increase of 0.5 and 1.4 percentage points over the baseline network, which was the largest improvement. Meanwhile, F1-score reached 0.91, indicating that the model performs well in both P and R, and demonstrated the strongest overall performance. In summary, the SEAM solution provides an optimized comprehensive performance enhancement, named YOLOv12-S.

### 3.2 The optimization of the up-sampling operator

Traditional up-sampling can restore image details and enhance image resolution through interpolation algorithms, but it may affect image quality. In response to the aforementioned issues, we used 5 up-sampling methods to compensate for potentially lost details and minimize the impact on image quality, and utilized 2 down-sampling methods for comparative analysis. We employed 5 up-sampling operators, namely wavelet feature upgrade (WFU), dynamic sampling (Dy-Sample), explicit visual center block (EVC), selective boundary aggregation (SBA), and CARAFE, and conducted a comparative analysis using 2 down-sampling operators, rectangular self-calibration module (RCM) and generalized-FPN (GFPN). Meanwhile, we selected 5 combination schemes that could improve performance individually. To validate algorithmic efficacy and investigate synergistic interactions among improvement modules, we performed comparative experiments with YOLOv12-S as the baseline. Inference time was measured on the forward pass only. Preprocessing steps such as image resizing and normalization, as well as postprocessing steps including Non-Maximum Suppression, were excluded from the reported timing results. Each model was run for 100 iterations after a 10-iteration warm-up, and the average forward pass latency is reported in milliseconds. Quantitative results are detailed in **Table 4**.

**Table 4:** Optimization results of different sampling operators.

Module	mAP@0.5 (%)	mAP@0.5:0.95(%)	Parameters	FLOPs(G)	Size (MB)	Inference time(ms)
YOLOv12-S	96.1	81.8	2653494	6.6	5.7	0.3
+WFU	95.8	79.3	3589110	8.3	7.6	0.4
+Dy-Sample	96.3	81.7	2665846	6.7	5.7	0.4
+EVC	96.4	81.8	4336118	11.7	9.1	0.9
+SBA	95.3	79.4	3144694	8.8	6.4	0.5
+CARAFE	96.6	82.5	2793926	6.9	6	0.6
+RCM	96.2	81.3	3056694	7.1	6.4	0.4
+GFPN	96.3	81.6	3108342	7.5	6.6	0.4
EVC+Dy-Sample	96.5	81.9	4228598	11.4	8.9	0.9
EVC+RCM	96.4	81.7	4722943	12.1	9.8	0.8
CARAFE+RCM	96.4	82.9	3197126	7.4	6.7	0.8
CARAFE+EVC	96	80.8	4356678	11.7	9.1	1.4
Dy-Sample+RCM	96.3	82.9	3069046	7.2	6.5	0.4

**Figure 7:**
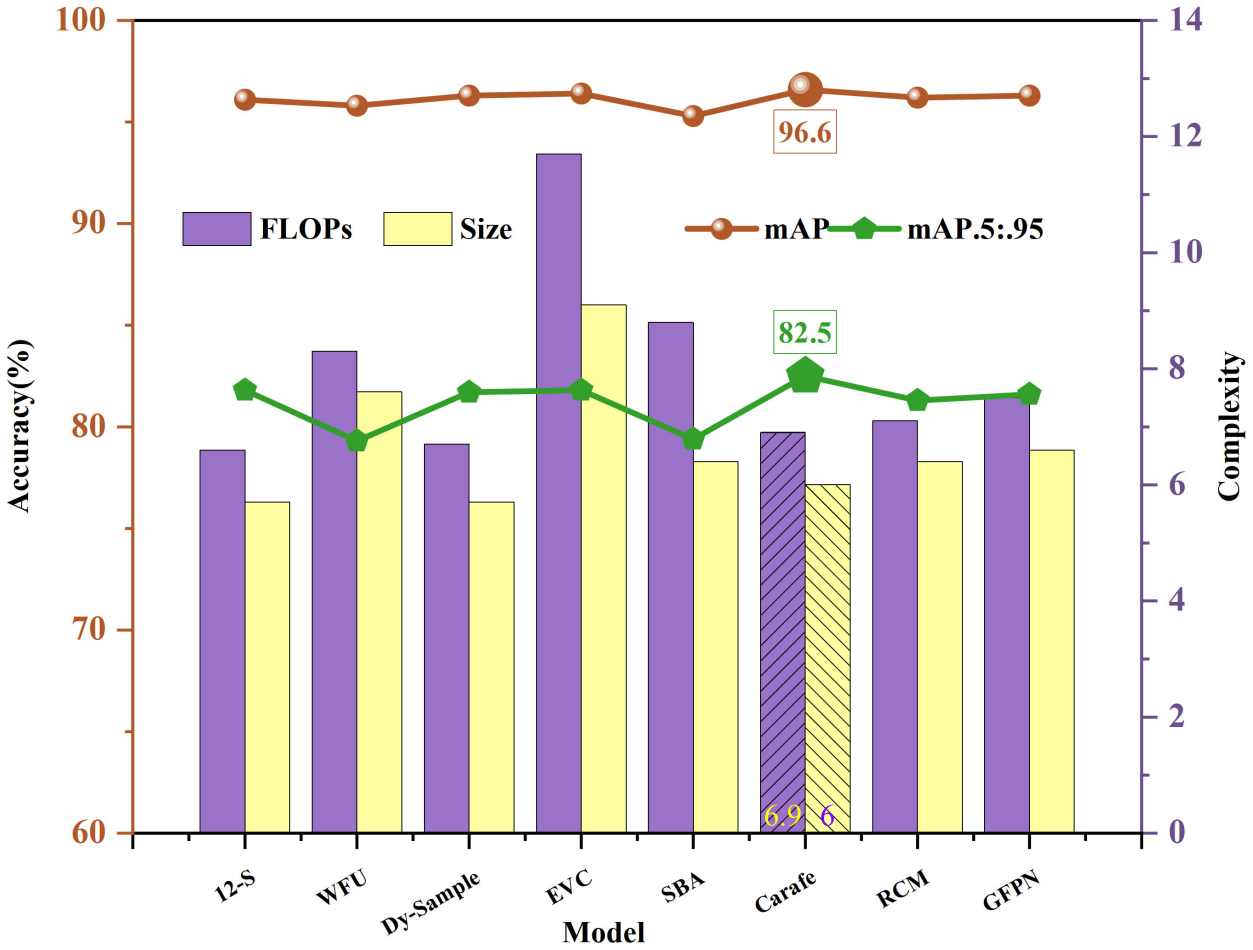
Comparison of accuracy and complexity of various sampling methods.

Among the 12 improvement schemes, the SEAM+CARAFE combination (YOLOv12-SC) outperformed the baseline network with mAP@0.5 and mAP@0.5:0.95 scores of 96.6% and 82.5%, respectively, achieving the greatest accuracy enhancement. The CARAFE module’s primary advantage resides in its capacity to aggregate contextual information within extensive receptive fields. Its kernel prediction module encodes and reorganizes compressed feature content, dynamically generating weights in a content-adaptive manner. The specific trends of each data are shown in **Figure 7**.

The above figure illustrates the variations in accuracy metrics (mAP@0.5 and mAP@0.5:0.95) and complexity metrics (parameters, FLOPs, and model size) across different improvement schemes. We can observe that the enhancement brought by the CARAFE scheme ranks only second, just above Dy-Sample (Liu et al., 2023). This indicates that the scheme achieves the maximum improvement in model accuracy (as indicated by the green and brown labels in the figure) at the cost of a relatively small increase in model complexity (represented by the purple and gold patterned bars in the figure).

Although RCM (Ni et al., 2024) and GFPN (Jiang et al., 2022), these two down-sampling methods, can expand the receptive field and acquire more features, they reduce the image resolution, which is prone to information loss, so the accuracy improvement is not significant. WFU (Li et al., 2024), SBA (Tang et al., 2023), these two up-sampling methods, perform well in addressing boundary blurring issues and restoring high-frequency details, and have advantages in target and localization aspects. However, they significantly impact model complexity, increase computational costs, and even lead to a decrease in accuracy.

### 3.3 The design of the detection head

The detection head is directly related to the accuracy and efficiency of object detection. Compared to traditional convolution, this paper proposed to introduce the PPA module, which incorporates a multi-branch strategy and adaptive feature enhancement, to construct a small target detection head with an attention mechanism. Additionally, four modules—Dynamic-Head (Han et al., 2024), MB Conv (mobile inverted residual bottleneck convolutional block), and ASFF (adaptively spatial feature fusion)—were introduced to evaluate the effectiveness of the enhanced detection head for small targets. Specific results are presented in **Table 5**.

**Table 5:** Results of different detector head improvements.

Module	AP(%)		P (%)	R (%)	mAP@0.5 (%)	mAP@0.5:0.95 (%)	Parameters	FLOPs (G)	Size (MB)
	Ripe	Unripe							
YOLOv12-SC	97.6	95.5	92.7	89.1	96.6	82.5	2793926	6.9	6
+PPA	97.6	96	93.0	89.2	96.8	83.7	3412650	9.9	7.3
+Dy-Head	97.6	95.5	92.1	90.1	96.6	81.5	2590942	5.5	5.6
+MB Conv	97.5	95.3	92	89.9	96.4	81.1	2473286	5.7	5.4
+ASFF	97.7	95.6	92.8	89.7	96.7	83.3	4487080	10.3	9.4
CARAFE+ASFF	97.5	95.6	92.4	89.9	96.6	83.2	4383592	10.1	9.2
SEAM+PPA	97.4	95.2	91.8	89.3	96.3	82.4	3272218	9.6	7.0

**Figure 8:**
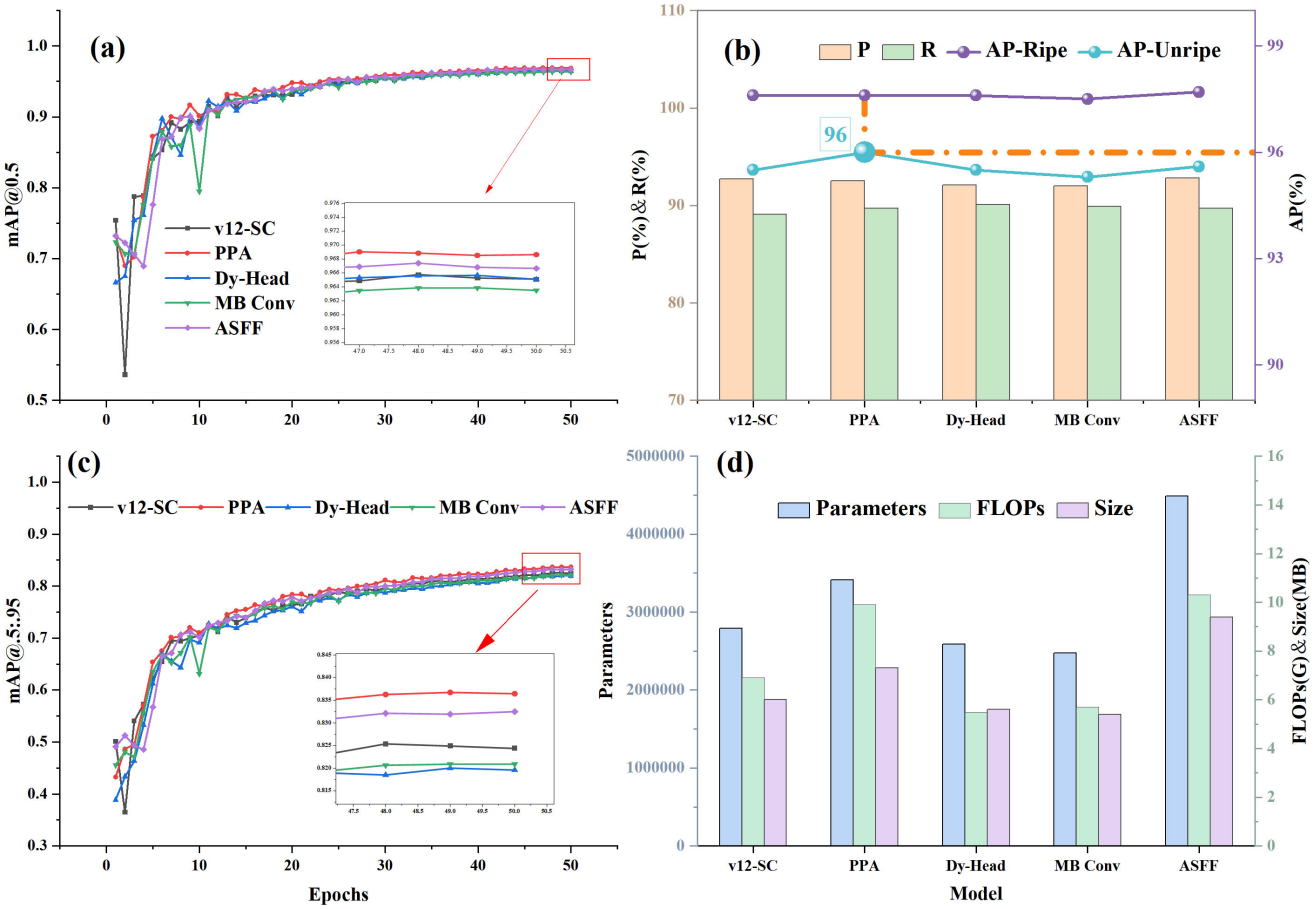
Effect of each detection head improvement scheme.

As evidenced in **Table 5**, although the model complexity of the detection head incorporating the Dy-Head module is slightly lower than that of the baseline network and the mAP@0.5 remains unchanged, the mAP@0.5:0.95 has decreased by 1 percentage point. The Dynamic Convolution module (where the dynamic fusion weight module is parameter-free) is based on the principle of dynamic coefficient generation, but its weight instability may disrupt feature consistency, compromising detection accuracy. If the MB Conv is used, the accuracy drop becomes more pronounced, attributed to the limited receptive field and the loss of feature information caused by the inverted bottleneck structure and depth-wise separable convolution. Both structures aim to build efficient and lightweight neural networks but are accompanied by varying degrees of accuracy loss. The specific data of each scheme are shown in **Figure 8**.

As can be seen from **Figure 8b**, although the accuracy of ripe fruits remains relatively stable at 97.6% (purple solid line), the accuracy of unripe fruits shows a significant increase (cyan solid line and orange dashed line), with Detect_PPA reaching a maximum of 96%. After integrating the PPA and ASFF modules into the detection head, both mAP@0.5 and mAP@0.5:0.95 have increased, but the overall model complexity with ASFF is higher than that with PPA (Parameters +31.48%, FLOPs +4.04%, Model Size +28.77%, **Figure 8d**).

Both modules improve small target detection performance. The core of the ASFF module lies in its adaptive feature fusion technology for weight allocation, while the PPA module further incorporates a multi-branch strategy to capture multi-scale features of objects, facilitating the interaction between local and global information. This approach not only compensates for the loss of small target features at a single scale but also improves the representational capability of small targets. In summary, among the four improved detection head methods, the PPA module demonstrates superior comprehensive performance in small target detection.

### 3.4 Ablation experiment

This paper takes the minimal-volume YOLOv12n as the baseline network. To address the three challenges of occluded targets, dense growth, and small target detection, improvements were implemented through the SEAM attention mechanism, the CARAFE up-sampling operator, and the attention-equipped PPA small target detection head, respectively. The specific data of the ablation experiments are presented in **Table 6**.

**Table 6:** Results of ablation experiments.

Module	SEAM	CARAFE	PPA	AP(%)		P (%)	R (%)	mAP@0.5(%)	mAP@0.5:0.95 (%)	FLOPs (G)	Size (MB)
				Ripe	Un-ripe						
YOLOv12n	×	×	×	96.9	94.3	92.6	88.3	95.6	81	6.0	5.4
YOLOv12-S	✓	×	×	97.2	95.1	92.5	89.4	96.1	81.8	6.6	5.7
YOLOv12-C	×	✓	×	97.4	94.8	92.1	89.5	96.1	81.5	6.7	5.7
YOLOv12-P	×	×	✓	97.4	95.0	91.7	89.1	96.2	82.1	9.4	6.8
YOLOv12-SC	✓	✓	×	97.6	95.5	92.7	89.1	96.6	82.5	6.9	6.0
YOLOv12-SP	✓	×	✓	97.6	95.2	91.8	89.3	96.3	82.4	9.6	7.0
YOLOv12-PC	×	✓	✓	97.5	95.4	92.2	89.2	96.5	82.4	9.7	7.1
YOLOv12-SCP	✓	✓	✓	97.6	96	93.0	89.2	96.8	83.7	9.9	7.3

√ and × represent the application or non-application of the improvement plan.

**Figure 9:**
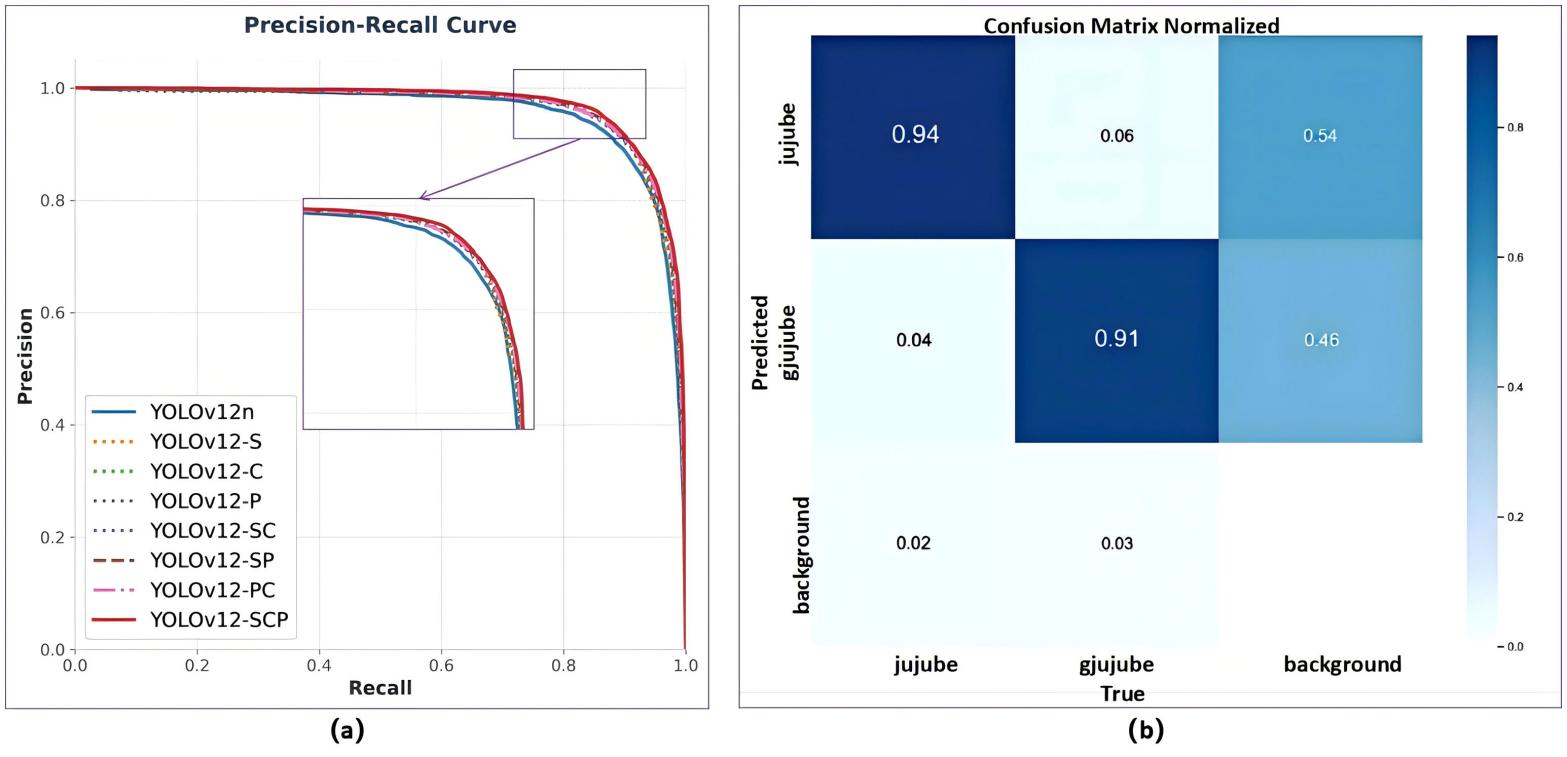
Results of ablation experiments. **(a)** P-R curves **(b)** confusion matrix normalized.

The improvement schemes in the above table are divided into three types: single improvement, dual improvement, and comprehensive improvement. Among single-module improvements, all three schemes increased precision, with Detect_PPA achieving the highest improvement (96.2% mAP@0.5 and 82.1% mAP@0.5:0.95). However, it also became the primary contributor to increased computational cost and model size (+56.67% FLOPs, +25.93% size). Similarly, in dual-module schemes, implementations incorporating the PPA module (SP, PC) and those excluding it (SC) similarly increased FLOPs and size by approximately 40.58% and 18.33%, respectively. To enhance the comprehensibility of the data, the information from the table above is visualized in a graph, as shown in **Figure 9**.

**Figure 9a** presents the P-R curves of various improvement schemes in the ablation experiment. The YOLOv12-SCP model has achieved satisfactory results in the identification of field flat jujube, not only demonstrating high detection accuracy but also maintaining a high recall rate (red solid line). **Figure 9b** displays the confusion matrix for the detection of field flat jujube, where the model’s recall rates for ripe and unripe fruits are 94% and 91%, respectively. The last column of the confusion matrix indicates the presence of false positives, primarily due to the model’s misidentification of some small, occluded, and unlabeled field flat jujube in complex environments. Multiple ablation experiments demonstrated that the integrated solution (YOLOv12-SCP) delivered optimal improvements, with mAP@0.5 and mAP@0.5:0.95 increasing by 1.2% and 2.7%, respectively, while mature and immature fruit detection accuracy rose by 0.8% and 1.7%, respectively.

**Figure 10:**
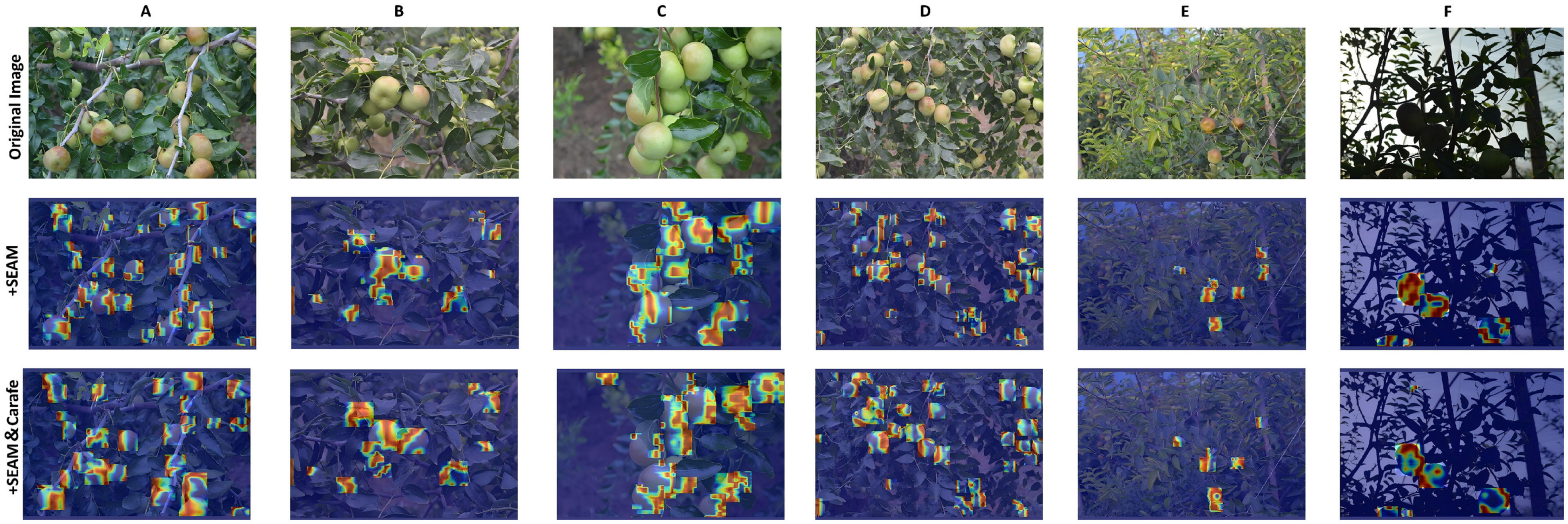
Results of Grad-CAM heatmaps.

To provide interpretability, we include Grad-CAM heatmaps (**Figure 10**) for selected intermediate layers. The heatmaps illustrate that the SEAM attention module helps the model focus more consistently on occluded fruit regions, while the CARAFE upsampling operator improves contextual inference compared to nearest-neighbor interpolation.

### 3.5 The comparison experiment of the mainstream one-stage algorithm

**Figure 11:**
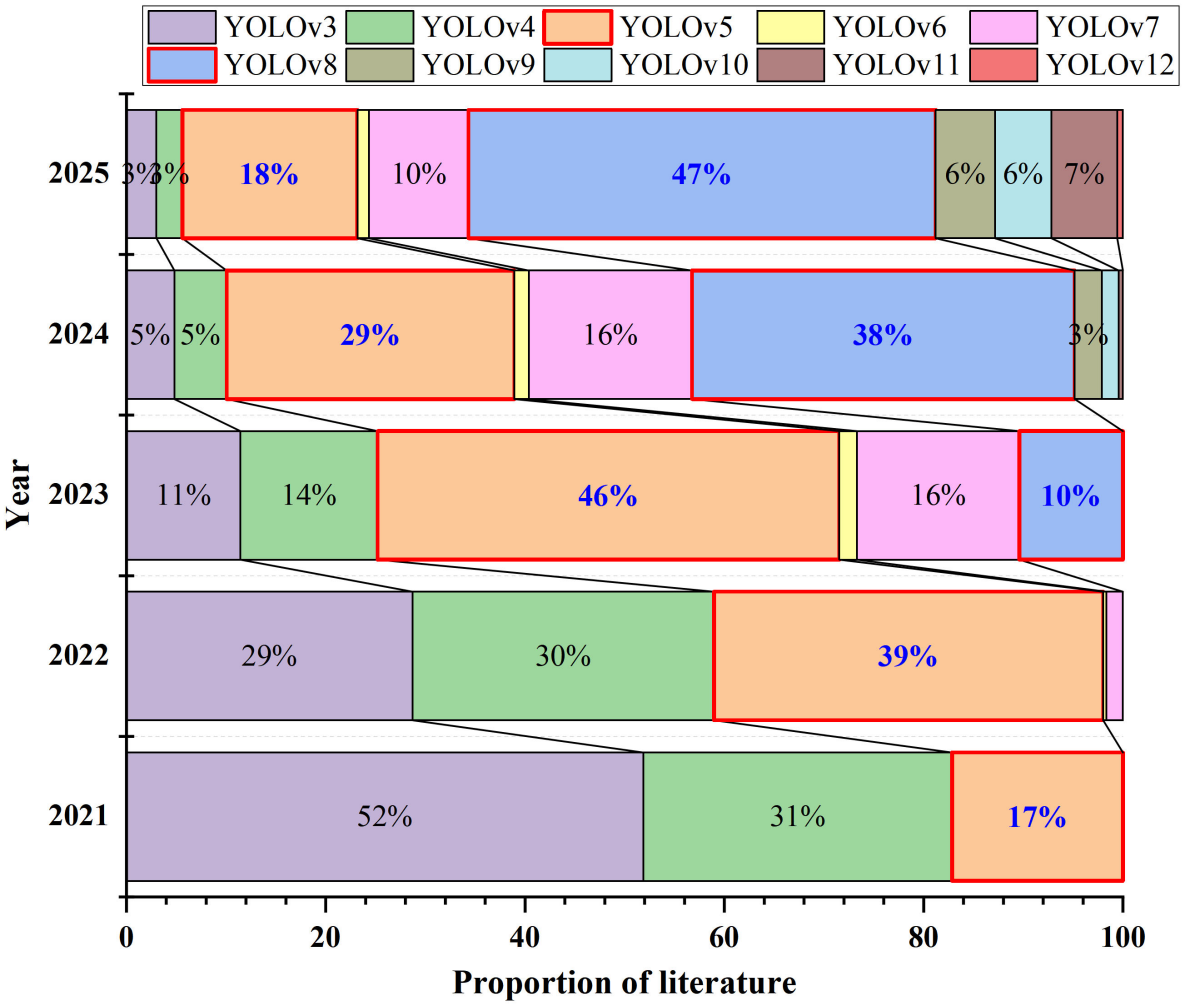
Statistical analysis of literature data from the past 5 years.

In 2020, YOLOv5, proposed by the Ultralytics team, propelled the YOLO series of networks into a period of rapid development. **Figure 11** details a statistical analysis of literature on YOLO from the past five years, conducted using the Web of Science and X-MOL databases.

As depicted in **Figure 11**, 2023 marked the explosive growth period of YOLOv5. With the development of the YOLO series networks, the utilization rate of YOLOv8 surpassed that of YOLOv5 in 2024. That is to say, in the past five years, the literature on the YOLO series networks has been predominantly focused on YOLOv5 and YOLOv8. Therefore, this paper will primarily conduct a horizontal comparative analysis of the improved algorithms of these two versions and the subsequently updated mainstream detection algorithms, with specific data presented in **Table 7**. Results are averaged over three runs (seeds 42, 2024, 2025) and reported as mean ± standard deviation. Training parameters and hyperparameter settings remained identical across all runs. The magnitude of the standard deviation reflects the stability of model convergence on this dataset.

**Table 7:** Experimental results of mainstream network comparison.

Module	AP(%)		P(%)	R(%)	mAP@0.5(%)	mAP@0.5:0.95(%)	F1 (%)	Parameters	FLOPs (G)	Size (MB)
	Ripe	Unripe								
YOLOv5n	96.6	93.0	91.3	86.4	94.8	73.4	89	1766623	4.2	3.7
YOLOv8n	97.3	95.1	91.2	88.1	95.8	80.5	90	3011238	8.2	6.2
YOLOv9-tiny	97.0	93.2	90.6	87.4	95.1	79.3	89	2165441	7.7	4.7
YOLOv10n	97.0	93.8	90.1	88.5	95.4	74.8	89	2707820	8.4	5.7
YOLOv11n	96.2	91.7	89.7	85.1	94.0	76.5	87	2590230	6.4	5.4
YOLOv12n	96.9	94.3	92.6	88.3	95.6	81.0	91	2520054	6.0	5.4
YOLOv12s	97.4	95.8	92.3	89.7	96.6	83.3	91	9097238	19.6	18.6
YOLOv12-SCP	97.6 ± 0.2	96.0 ± 0.3	93.0 ± 0.4	89.2 ± 0.5	96.8 ± 0.2	83.7 ± 0.4	91 ± 0	3412650	9.9	7.3

**Figure 12:**
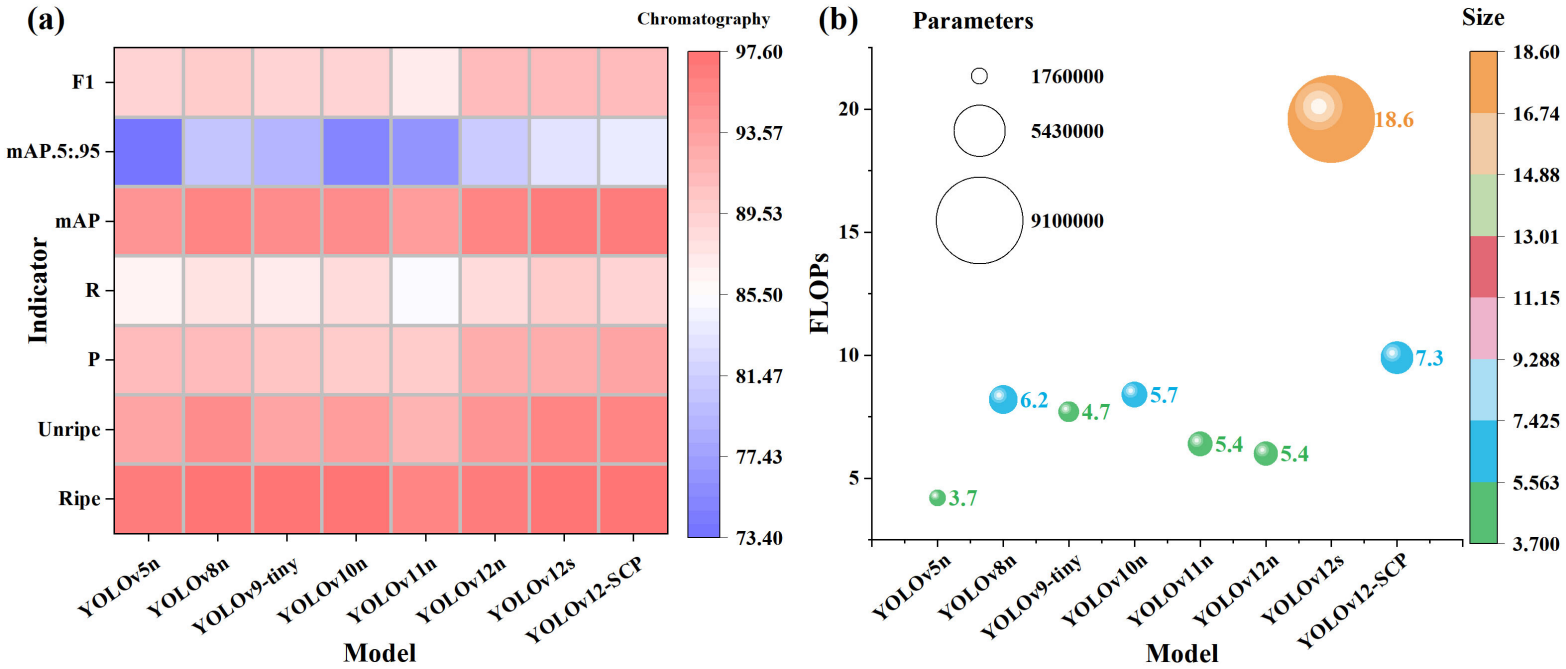
Results of comparative experiments on mainstream networks. **(a)**: accuracy comparison results **(b)**: complexity comparison results, the size of the spheres represents the quantity of parameters, the color represents the size of the models, and the position represents the value of FLOPs.

By initially comparing models with n-scale, we observed that YOLOv8n achieves the highest accuracy (mAP@0.5 = 95.8%), but its complexity metrics (Parameters, FLOPs, and Model) are all higher than those of YOLOv12n, which ranks second in accuracy (mAP@0.5 = 95.6%). Therefore, we selected YOLOv12n as the baseline network for further improvements. Experimental results demonstrated that the improved YOLOv12-SCP network achieved 1.2% higher accuracy than the baseline, despite increased complexity. Comparative analysis with the same-series s-scale model (YOLOv12s) further revealed superiority of YOLOv12-SCP: 0.2% higher accuracy accompanied by 62.49% fewer parameters, 49.49% lower FLOPs, and 60.73% reduced model size. This indicates that the accuracy of YOLOv12-SCP, based on the n-scale, is even slightly higher than that of the s-scale model, while the complexity metrics are significantly reduced. To more intuitively enhance the expressiveness of the data, we have divided the data from the above table into accuracy and complexity for visual comparative analysis, as shown in **Figure 12**.

According to **Figure 12a**, the color of all accuracy metrics for YOLOv12s and YOLOv12-SCP are almost identical (the last two columns), and both are darker than those of other networks, indicating that these two networks have the highest accuracy. Meanwhile, in **Figure 12b**, it is evident that the parameters and model size of YOLOv12s are significantly larger (orange sphere) than those of all other networks of the same scale, further demonstrating the superior comprehensive performance of the improved network. Collectively, this method achieves superior detection accuracy for occluded small targets in complex agricultural scenes (surpassing even small-scale models), while maintaining model complexity and size comparable to nano-scale counterparts, ultimately delivering optimal overall performance.

**Figure 13:**
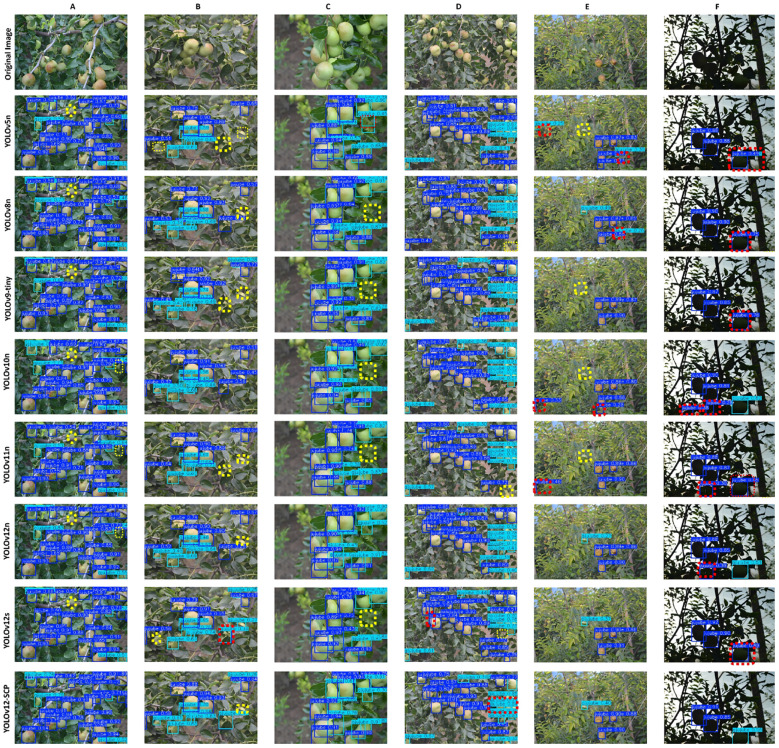
Detection effect diagrams of various networks. **(A)** branch occlusion. **(B)** leaf occlusion. **(C)** fruit overlap. **(D)** small target detection. **(E)** heavy occlusion. **(F)** low light. Yellow dashed box: missed detection. Red dashed box: false detection.

It should be noted that, although the dataset used in this study was collected from a single orchard during a single harvest season, the images cover multiple times of day (morning, midday, and late afternoon) as well as varying weather conditions, including both sunny and overcast skies. In subsequent analysis, we observed that the model maintained consistent detection performance across subsets with markedly different illumination levels. This consistency offers preliminary evidence of the method’s adaptability under the visual conditions encountered at this site. This paper compared the detection performance of the improved network YOLOv12-SCP with 7 networks (YOLOv5n, YOLOv8n-v12n, and YOLOv12s), as shown in **Figure 13**.

Based on **Figure 13**, all networks exhibit certain levels of missed detection and false detection, with missed detection being more prevalent than false detection. The primary reason for this is the weak feature representation caused by small targets and the feature loss due to complex occlusions. In **Figure 13A**, it is observed that there is a fruit (marked by a yellow solid-line circle) that only the improved network can detect, while all other networks miss it. The potential reason is that the SEAM+CARAFE module in YOLOv12-SCP compensates for the feature information loss in occluded areas and combines global and local information for prediction. Similarly, in **Figure 13C**, missed detection occur due to leaf occlusions and overlapping fruits. Besides occlusion, another potential reason is the close color similarity between the fruits and the leaf background (leaf green), which increases the detection difficulty. Although the proposed method achieves overall performance gains, certain limitations persist under extreme field conditions. For example, when jujube fruit is more than 70% occluded by leaves or located in deeply shadowed areas, the model still produces missed detections (see **Figure 13B, D**). This suggests that spatial information from a single RGB frame may not be sufficient to handle the most severe occlusion scenarios. Future work could explore the use of temporal information or multi−view fusion to further improve robustness in these challenging cases. Overall, the improved network demonstrates superior overall detection performance.

## 4 Discussion

The objective of this study is to improve the detection accuracy for small objects under occluded conditions. This paper addresses the challenges of overlapping occlusion and small target detection of flat jujube in complex agricultural environments by proposing a YOLOv12n-based field flat jujube detection method—YOLOv12-SCP. Numerous researchers have investigated occluded small object detection in agricultural contexts. For instance, Liang et al. (2025) develop a cherry tomato detection method based on YOLOv8, incorporating an attention-driven dynamic detection head to achieve an accuracy of 95.3%. For blueberry detection, a task that likewise falls under the umbrella of occluded small object detection, Mu et al. (2025) conduct a benchmark analysis of YOLO series variants spanning YOLOv8-v12. Their results demonstrate that YOLOv12m delivers the highest detection accuracy at 93.3%. YOLOv12-SCP achieved 96.8% and 83.7% in mAP@0.5 and mAP@0.5:0.95, respectively. Ripe and unripe fruits achieved AP values of 97.6% and 96.0%, respectively. This paper comprehensively compares the improved network with other mainstream single-stage object detection networks, demonstrating its comprehensive advantages in accuracy and complexity through heat-map and bubble charts, respectively, and verifies their detection performance.

This research introduces a SEAM attention mechanism designed to enhance feature representation in non-occluded regions while compensating for the feature degradation in occluded areas (Yu et al., 2024). Adjusting the kernel sizes of the three parallel branches in CSMM yields denser multi-scale sampling and stronger contextual reasoning, verifying its compensation for occlusions. Building on this foundation, the CARAFE operator further expands the effective receptive field while dynamically assigning higher weights to small target regions through a upsampling kernel. This dual approach effectively preserves essential details while suppressing irrelevant redundant information. Finally, to address the challenges of limited information and background confusion in small object detection, this study introduces a PPA small object detection head that integrates multi-branch fusion and adaptive feature enhancement (Xu et al., 2024). By combining its parallel small-scale branches with 3 larger-scale continuous branches from the SEAM, the head strengthens local feature representation for small objects. Furthermore, its attention mechanism, coupled with the CARAFE operator (Guo et al., 2025), effectively reduces the miss detection rate.

Despite its significant accuracy advantages, the YOLOv12-SCP model exhibits certain limitations that warrant consideration. The dataset originates from a single orchard and a single growing year. While random splitting provides an objective evaluation of the model on the available data, it does not yet assess how well the method generalizes to different orchards, varying planting densities, or different growing seasons. The visual appearance of flat jujube fruit in the field may shift with orchard management practices and regional climate differences. Therefore, cross−orchard and cross−season external validation remains an important direction for future work. The dataset constructed in this paper and the task−specific integration strategy proposed here establish a methodological foundation for such subsequent investigations. Additionally, the model’s complexity and detection efficiency require further optimization. Future research may explore lightweight and real-time optimization of models through methods such as structured pruning, knowledge distillation, and model quantization. Meanwhile, we can reduce detection time and improve detection efficiency through methods such as attention mechanisms, structural adjustments, and optimization of post-processing procedures. To conclude, YOLOv12-SCP provides a high-precision solution for detecting small, occluded targets and offers a practical foundation for further research into lightweight and real-time network improvements.

## 5 Conclusion

This paper addresses the challenges of overlapping occlusion and small target detection of flat jujube in complex agricultural environments by proposing a YOLOv12n-based field flat jujube detection method—YOLOv12-SCP. The approach addresses occlusion and small−target challenges through a combination of redesigned SEAM attention branches, CARAFE upsampling, and a PPA-based detection head. Experimental results on a self-constructed field dataset demonstrate clear improvements in detection accuracy under realistic agricultural conditions. The proposed improved offers a practical solution for automated yield estimation and robotic harvesting applications.

## Data Availability

The data analyzed in this study is subject to the following licenses/restrictions: Agricultural products. Requests to access these datasets should be directed to Shilin Li, lsl6080@sxau.edu.cn.
